# Interventions to reduce post-acute consequences of diarrheal disease in children: a systematic review

**DOI:** 10.1186/s12889-018-5092-7

**Published:** 2018-02-01

**Authors:** Patricia B. Pavlinac, Rebecca L. Brander, Hannah E. Atlas, Grace C. John-Stewart, Donna M. Denno, Judd L. Walson

**Affiliations:** 1 Department of Global Health, University of Washington, Seattle, WA USA; 2 Department of Epidemiology, University of Washington, Seattle, WA USA; 3 Department of Pediatrics, University of Washington, Seattle, WA USA; 4 Department of Medicine (Infectious Disease), University of Washington, Seattle, WA USA; 5 Department of Health Services, University of Washington, Seattle, WA USA

**Keywords:** Pediatric diarrhea management, Child growth, Diarrhea interventions, Child mortality, Long-term sequelae of diarrhea

## Abstract

**Background:**

Although acute diarrhea often leads to acute dehydration and electrolyte imbalance, children with diarrhea also suffer long term morbidity, including recurrent or prolonged diarrhea, loss of weight, and linear growth faltering. They are also at increased risk of post-acute mortality. The objective of this systematic review was to identify interventions that address these longer term consequences of diarrhea.

**Methods:**

We searched Medline for randomized controlled trials (RCTs) of interventions conducted in low- and middle-income countries, published between 1980 and 2016 that included children under 15 years of age with diarrhea and follow-up of at least 7 days. Effect measures were summarized by intervention. PRISMA guidelines were followed.

**Results:**

Among 314 otherwise eligible RCTs, 65% were excluded because follow-up did not extend beyond 7 days. Forty-six trials were included, the majority of which (59%) were conducted in Southeast Asia (41% in Bangladesh alone). Most studies were small, 76% included less than 200 participants. Interventions included: therapeutic zinc alone (28.3%) or in combination with vitamin A (4.3%), high protein diets (19.6%), probiotics (10.9%), lactose free diets (10.9%), oral rehydration solution (ORS) formulations (8.7%), dietary supplements (6.5%), other dietary interventions (6.5%), and antimicrobials (4.3%). Prolonged or recurrent diarrhea was the most commonly reported outcome, and was assessed in ORS, probiotic, vitamin A, and zinc trials with no consistent benefit observed. Seven trials evaluated mortality, with follow-up times ranging from 8 days to 2 years. Only a single trial found a mortality benefit (therapeutic zinc). There were mixed results for dietary interventions affecting growth and diarrhea outcomes in the post-acute period.

**Conclusion:**

Despite the significant post-acute mortality and morbidity associated with diarrheal episodes, there is sparse evidence evaluating the effects of interventions to decrease these sequelae. Adequately powered trials with extended follow-up are needed to identify effective interventions to prevent post-acute diarrhea outcomes.

**Electronic supplementary material:**

The online version of this article (10.1186/s12889-018-5092-7) contains supplementary material, which is available to authorized users.

## Background

Close to 600,000 children die each year from diarrheal disease, the majority in low- and middle-income countries (LMICs) [1]. Children with a single episode of moderate-to-severe diarrhea (MSD) experience an 8.5-fold higher risk of dying in the 60-days following the episode compared to age-matched healthy children, despite standard diarrhea case management including rehydration and zinc [2]. A verbal autopsy study conducted in 7 LMICs found that 55.6% of pediatric diarrhea deaths occurred in children who had been rehydrated [3]. Although rehydration and zinc have resulted in millions of lives saved from diarrhea, they may be insufficient to prevent all diarrhea-associated mortality. 

The consequences of diarrhea extend beyond acute dehydration and electrolyte imbalance. Over two-thirds of deaths associated with diarrhea occur more than 7 days after presentation [2]. An episode of MSD is also associated with subsequent loss of length/height-for-age z-score (LAZ/HAZ), a measure of chronic malnutrition [2, 4]. Undernutrition is linked to half of all diarrhea-associated mortality and is associated with other long-term outcomes including reduced school attendance and future earning potential [2, 5, 6].

While mortality from diarrheal diseases has declined since the 1990’s, incidence rates have remained stable and there is increasing recognition of the morbidity, disability, and long-term consequences associated with diarrhea. We conducted a systematic review to identify and summarize randomized controlled trials (RCTs) of diarrhea management interventions to determine effects on death, anthropometric status, and prevalence and incidence of diarrhea in the post-acute period.

## Methods

The systematic review followed PRISMA guidelines. We searched Medline for English-language RCTs published between January 1, 1980 and October 31, 2016 conducted among children under 15 years of age presenting with diarrhea (all diarrhea definitions accepted) at the time of treatment. Specifically, we searched for trials evaluating 1 of the following interventions: antiemetics, antibiotics, antiprotozoals, antisecretories, dietary supplements, intravenous hydration therapy, oral rehydration therapy, probiotics, prebiotics, lactose replacement, and therapeutic zinc. These interventions were chosen based on consultation with experts in the field. The search terms used were as follows:

((((((((((((antibiotic OR antiinfective OR anti-infective OR antimicrobial OR antiparasitic OR anti-parasitic OR antiprotozoa* OR anti-protozoa* OR ciprofloxacin OR erythromycin OR metronidazole OR antiemetic* OR anti-emetic OR anti-vomit* OR antidiarrheal OR secretoinhibit* OR antipropulsive OR antisecret* OR anti-secret OR breast* OR formula* OR milk OR wean* OR treatment OR management OR “amylose maize starch” OR hams OR lams OR prebiotics OR “resistant starch” OR bifidobacter* OR lactobacill* OR lactococc* OR microbi* OR probiotic* OR fluid OR intravenous OR IV OR ORS OR “oral rehydration salt” OR ORT OR “oral rehydration therapy” OR polymer OR rehydration OR minerals OR zinc)))) AND (“1980/01/01”[Date - Publication]: “2016/10/31”[Date - Publication])) AND (((“bloody stool” OR diarrh* OR dysentery OR gastroenterit*))))) AND ((((clinical trial) OR placebo-controlled trial) OR randomized controlled trial))))) NOT cancer) NOT antibiotic associated diarrhea)

Filters: Clinical Trial; Humans; English; Child: birth-18 years

We excluded studies in 2 steps. The first step aimed to exclude trials that did not address the populations or interventions of interest. Specifically, studies conducted in high-income countries (as defined by the World Bank as of June 2015) [7], those that did not include children with diarrhea at enrollment, utilized a design other than an RCT, or did not present individual-level outcome data were excluded in the first round. The second round excluded trials with insufficient follow up (less than 7 days) and those that lacked outcome data on mortality, length/height, LAZ/HAZ, weight, weight-for-age z-score (WAZ), weight-for-height z-score (WHZ), mid upper arm circumference (MUAC), or diarrhea presence at a pre-specified follow-up point ≥7 days after enrollment. Although weight may be misclassified during diarrhea illness due to fluid loss, in the context of an RCT, groups were assumed to be balanced with regard to hydration status. Therefore, weight, WAZ, and WHZ were considered valid outcomes. Diarrhea duration (other than presence of diarrhea at a pre-specified time point beyond 7-days) and stool output were not included as outcomes because they were considered intermediate to the outcomes of interest in this review.

All titles and abstracts were screened by 2 reviewers (PBP and HEA) and abstracts of agreed-upon titles were examined for inclusion. Full texts of agreed-upon abstracts were reviewed for inclusion by RLB and HEA with final input from PBP. The following study-specific information was abstracted from included trials: intervention, control group, population, dates of enrollment, sample size, duration of follow up, reported outcomes, and data on effect sizes of relevant outcomes, and associated confidence intervals (CIs). Details on data abstraction and calculations are provided in the supplementary material for this manuscript (Additional file 1).

A modified Grading of Recommendations Assessment Development and Evaluation (GRADE) approach was developed to assess study design elements including sample size, number of participants lost to follow up or withdrawn from the study, and blinding and allocation concealment methods to evaluate the quality of studies. We did not assess the GRADE elements of directness or consistency, as these elements are specific to results reported within a given intervention and outcome category and this review assessed multiple interventions and outcomes. All trials started with 4 points because all were randomized controlled trials and 1 point was deducted for each of the following elements: sparse data (< 200 trial participants), > 5% loss-to-follow-up or withdraws, or lack of double-blinding. Reviewers (HA and RB) applied the modified GRADE system included in this review and categorized each study as high quality (4 points), moderate (3 points), low (2 points), or very low (1 point) based on their final score. In addition to the elements required for GRADE, from included trials we abstracted whether or not a primary endpoint was declared (and whether the primary endpoint was 1 of the endpoints included in this review) and any mention of power calculations for included outcomes.

## Results

**Fig. 1:**
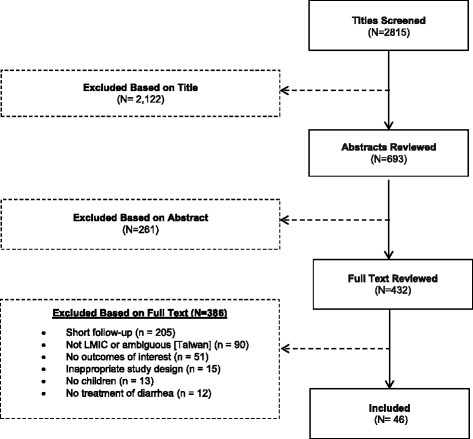
Flow chart of included trials of diarrhea management interventions

The Medline search returned 2815 titles, of which 693 abstracts and 432 full texts were reviewed, and 385 excluded based on full-text review (Fig. 1). Among the 314 studies that were eligible based on study location, design, and population (included based on first exclusion step), most (205 [65.2%]) were excluded for failure to meet our criteria for length of follow up (7 days or more) and 51 (16.2%) were excluded because no outcomes of interest were reported in the second exclusion phase.

Forty-six trials were included in this review, the majority (27 studies [58.7%]) of which were conducted in the WHO-classified Southeast Asia region [8–34], with 19 (70.4%) conducted in Bangladesh alone (Table 1). Seven studies (15.2%) were conducted in the Americas [35–41], 7 studies (15.2%) reported data from Africa [42–48], 2 from the Eastern Mediterranean (Pakistan) [49, 50] and 2 from Europe (Turkey) [51, 52]. One study was conducted in 3 countries (Ethiopia, Pakistan, and India) [53]. The majority of the trials were conducted in inpatient settings (35 studies, 76.1%). Five (10.9%) trials were community-based, and the remaining 6 (13.0%) were conducted in outpatient settings. The most common interventions included therapeutic zinc (15 studies [32.6%], 2 of which was assessed in the same trial as vitamin A), and high protein diets (9 studies [19.6%]). Probiotics were assessed in 5 studies (10.9%), and 5 trials evaluated lactose-free diets (10.9%). Four were trials of ORS formulations (8.7%), and 3 (6.5%) trials evaluated dietary supplements, including dietary fiber (2 studies), and glutamine (1 study). Three (6.5%) trials were of other dietary interventions, a semi-elemental diet and 2, 3-armed trials evaluated ready-to-use therapeutic food (RUTF) or micronutrient powder. Only 2 (4.3%) of the trials that fit our inclusion criteria evaluated antimicrobial treatments (1 antibiotic and 1 antiprotozoal). We did not find any trials of intravenous (IV) rehydration, antisecretory agents, or antiemetic agents that met our inclusion criteria. The sample size of included studies ranged from 18 to 8070 and only 11 (23.9%) of the 46 trials included more than 200 participants (1 ORS, 2 probiotic, 2 RUTF/micronutrient, 1 vitamin A + zinc, and 5 zinc trials). Just over half of included trials (52.2%) reported power/sample size calculations, the majority of which (11 [48%] of the 23) were powered for the outcome of diarrhea duration/stool output, outcomes not included in this systematic review.

**Table 1:** Characteristics of included studies

Reference [Ref #]	Country	Population	Intervention	Comparator	Number with follow up data	Pre-specified follow up time	Relevant outcomes measured and power^i^	Modified GRADE score
Antimicrobial Intervention								
Gilman 1980 [8]	Bangladesh	Inpatient adults and children with blood, pus cells, and mucus in stool, 4 or more stools/day, and culture-confirmed *Shigella* infection	Low-dose ampicillin (50 mg/kg/day)	High-dose ampicillin (150 mg/kg/day)	56 children	21 days	Mortality (power/sample size calculations not reported)	Very low^Ω,ΦΨ^
Amadi 2002 [42]	Zambia	Inpatient children 12–85 mo with diarrhea (at least 3 stools per day that take the shape of a container or can be poured) and *Cryptosporidium* oocysts	5 mL of 20 g/L nitazoxanide oral suspension, twice daily for 3 days	Placebo	96	8 days	Mortality (powered for outcome of clinical response)	Moderate^Ω^
Dietary Supplements								
Alam 2000 [26]	Bangladesh	Inpatient males 4–18 mo with acute non-dysenteric diarrhea	WHO ORS with dietary fiber(20 g/L Benefiber®)	Standard WHO-ORS	150	7 days	Weight gain at day 7 from enrollment (powered for the outcome of stool output)	Moderate^Ω^
Rabbani 2001 [15]	Bangladesh	Severely malnourished (< 60% NCHS standard), inpatient male infants 5–12 mo with persistent diarrhea (> 3 loose stools/day for 14 days), treated with ciprofloxacin	Rice-based diet with dietary fiber (250 m/L cooked, green banana, 7 days; or rice-based diet with 1 g/kg to 8 g/kg of pectin supplement) 7 days	Calorically equivalent control diet: rice-based diet only, 7 days	62	At least 7 days, or until end of diarrhea	Proportions recovered from diarrhea [formed stool] at days 7, 8, 9, and 10 (powered for outcome of diarrhea recovery duration)	Moderate^Ω^
Yalcin 2004 [51]	Turkey	Inpatient children 6–24 mo with acute diarrhea	Glutamine supplement - 0.3 g/kg/day, for 7 days	Placebo	143	3 months	Weight gain at day 30, 60, and 90 from enrollment (powered for the outcome of diarrhea duration)	Low^Ω, ϑ^
High Protein Diets								
Datta 1990 [9]	India	Inpatient children under 5 y/o with acute dysentery (visible blood and mucus in stools), treated with nalidixic acid	Extra servings of milk (30% of total daily caloric requirements), in addition to standard hospital diet	Standard hospital diet	96	15 days	Weight and MUAC at day 15 (power/sample size calculations not reported)	Very low^Ω, Φ, ϑΨ^
Kabir 1992 [10]	Bangladesh	Inpatient children 2–4 y/o with culture-confirmed *Shigella* dysentery, treated with nalidixic acid or other microbial	High protein diet (15% of total energy from protein), 21 days	Standard diet (7.5% of total energy from protein), 21 days	22	21 days	Change in weight, height, WAZ, WHZ, HAZ, MUAC, and triceps skinfold thickness at day 21 from admission (power/sample size calculations not reported)	Very low^Ω, Φ, ϑΨ^
Kabir 1993 [11]	Bangladesh	Outpatient children 2–5 y/o with acute diarrhea and culture-confirmed *Shigella* spp., treated with nalidixic acid or pivmecillinam	High protein diet (15% of total energy from protein), 21 days	Standard diet (7.5% of total energy from protein), 21 days	69	21 days	Change in WHZ, WAZ, and HAZ at day 21 from admission (power/sample size calculations not reported)	Very low^Ω, Φ, ϑΨ^
Mazumder 1997 [12]	Bangladesh	Malnourished (< 80% NCHS median), inpatient children 12–48 mo with bloody or bloody mucoid diarrhea and culture-confirmed *Shigella*, treated with nalidixic acid	High calorie and high protein diet, 4960 kJ/l for 10 days	Standard hospital diet, 2480 kJ/l for 10 days	75	40 days	Percent change in WAZ and WHZ at 10, and 40 from admission (power/sample size calculations not reported)	Low^Ω, Φ, Ψ^
Nurko 1997 [36]	Mexico	Inpatient children 3–36 mo with persistent diarrhea (3 or more loose stools/day for 14 days or longer) and third-degree malnutrition (< 60% NCHS median), treated with TMP-SMX or metronidazole	High protein diet (2 groups: chicken-based diet, or soy-based diet)^ii^	Standard cultural dietary treatment for diarrhea (elemental diet or “Vivonex”)	41	16 days minimum	Weight at end of intervention and at discharge; proportion with nutritional recovery^iii^ at end of intervention (powered for outcome of diarrhea duration)	Very low ^Ω, ϑ, Ψ^
Kabir 1998 [13]	Bangladesh	Inpatient children 2–60 mo with acute bloody mucoid diarrhea, treated with nalidixic acid or pivmecillinam	High protein diet (15% of total energy from protein), 21 days	Standard protein diet (7.5% of total energy from protein), 21 days	59	6 months post- intervention	Change in weight, height, WHZ, WAZ, and HAZ at 6 months compared to post-intervention measurements (power/sample size calculations not reported)	Very low ^Ω, Φ, ϑ, Ψ^
Mazumder 2000 [14]	Bangladesh	Malnourished (< 80% of NCHS median) inpatient children 12–48 mo with acute bloody or mucoid diarrhea, treated with nalidixic acid of pivmecillinim	High calorie & protein milk-cereal formula (4960 kJ/l), 10 days	Control milk-cereal formula (2480 kJ/l), 10 days	75	10 days	Percent change in WAZ at day 10, compared to admission WAZ (power/sample size calculations not reported)	Very low^Ω, ϑ, Ψ^
Valentiner-Branth 2001 [43]	Guinnea-Bissau	Community-based children under 3 y/o with persistent diarrhea per mother’s report	Counseling on the importance of breastfeeding and of a nutritious diet, and a high protein millet gruel with a multivitamin tablet (including zinc), until the end of a 7 day period without diarrhea	Counseling on the importance of breastfeeding and of a nutritious diet	101	9 months	Difference in knee-heel length, height and weight at end of intervention and day 90, compared to admission measurements (powered for outcome of diarrhea duration)	Very low^Ω, Φ, Ψ^
Rollins 2007 [44]	South Africa	Inpatient HIV+ children 6–36 mo with persistent diarrhea (4 or more loose or watery stools/day, for 5 days or more)	Enhanced nutritional support: standard nutritional support + extra protein to provide 150 kcal/kg/day and 4.0–5.5 g protein/kg/day (as milk or powdered protein, depending on age), until diarrhea resolved	Standard nutritional support: maize porridge + milk formula, until diarrhea resolved	104	26 weeks	Mortality, median change in weight-SDs ^iv^and WAZ at 26 weeks; proportions underweight (WAZ < − 2 SDs) and stunted (LAZ < − 2 SDs) at 26 weeks (powered for outcome of weight change)	Very low^Ω, Φ, ϑ, Ψ^
Lactose Free Diet								
Bhan 1988 [16]	India	Outpatient children 3–24 mo	Legume and cereal-based formula (lactose-free), until recovery or a minimum of 7 days	Calorically equivalent milk-based formula, until recovery or a minimum or 7 days	57	At least 7 days, or until end of diarrhea	Weight gain at day 7 and at recovery compared to admission weight (power/sample size calculations not reported)	Very low^Ω, Φ, ϑ, Ψ^
Bhutta 1991 [50]	Pakistan	Outpatient males 6 mo - 3 yo with persistant diarrhea (increased frequeny and reduced consistency lasting 2 weeks or more)	Soy milk (lactose-free) for 7 days, followed by khitchri and yogurt for 7 days	Khitchri and yogurt for 14 days	73	14 days	Weight gain at day 7 and 14 compared to admission weight (power/sample size calculations not reported)	Very low^Ω, Φ, ϑ, Ψ^
Lozano 1994 [38]	Colombia	Inpatient children 1–24 mo with diarrhea (4 or more watery stools in a 24 h period) and dehydration	Lactose-free feeding formula, 21 days	Feeding formula with lactose, 21 days	52	6 weeks post-discharge	Weight increment at 6 weeks (powered for outcomes of diarrhea duration)	Very low^Ω, Φ, ϑ, Ψ^
Bhatnagar 1996 [17]	India	Inpatient \children 3–24 mo, with persistent diarrhea (3 or more liquid stools/day for 14 days)	Puffed rice cereal, sugar, oil, and milk protein, 120 h	Puffed rice cereal, sugar, oil, and egg white protein (lactose-free), 120 h	116	4–6 weeks after discharge	Proportion of patients whose weight on day 7 was lower than at rehydration; probability of continuing diarrhea at each day to day 12 (power/sample size calculations not reported)	Very low^Ω, Φ, Ψ^
de Mattos 2009 [37]	Brazil	Inpatient male infants 1–30 mo with persistent diarrhea (3 or more liquid stools per day for 14 days)	Amino-acid based diet or soy-based diet, or hydrolyzed protein-based diet^v^	Yogurt-based diet	154	7 days post-discharge	Difference in weight gain and change in WHZ at discharge compared to admission measurements (powered for outcomes of stool output and diarrhea duration)	Very low^Ω, Φ, ϑ, Ψ^
Other Dietary Interventions								
Eichenberger 1984 [35]	Brazil	Inpatient infants 1–11 mo with acute to subacute gastroenteritis with diarrhea	Semi-elemental diet with low osmolarity and high content of hydrolyzed lactalbumin	Standard hospital diet	38	21 days	Weight at day 21 compared to weight at beginning of therapy (power/sample size calculations not reported)	Very low ^Ω, Φ, ϑ, Ψ^
van der Kam 2016 [45]	Uganda	Non-malnourished, outpatient children 6–59 mo with diarrhea (3 or more loose stools [bloody or nonbloody] per 24 h by mothers’ report), malaria, or lower respiratory tract infections	Ready-to-use Therapeutic Foods (RUTF), plus instructions to feed the child an extra meal/day for 14 d; or micronutrient powder plus instructions to feed the child an extra meal/day for 14 d	An instruction to feed the child an extra meal/day for 14 d	941 with diarrhea only	6 months	Incidence of WHZ < − 2, MUAC < 115 mm, or nutritional oedema during follow up (powered for combined outcome of negative nutritional outcome)^vi^	Low ^ϑ, Ψ^
van der Kam 2016 [46]	Nigeria	Non-malnourished or moderately malnourished outpatient children 6–59 mo with diarrhea (3 or more loose stools [bloody or nonbloody] per 24 h by mothers’ report), malaria, or lower respiratory tract infections	Ready-to-use Therapeutic Foods (RUTF), plus instructions to feed the child an extra meal/day for 14 d; or micronutrient powder plus instructions to feed the child an extra meal/day for 14 d	An instruction to feed the child an extra meal/day for 14 d	1171 with diarrhea only	6 months	*For non-malnourished children at enrollment*: Incidence of WHZ < − 2, MUAC < 115 mm, or nutritional oedema during follow up. *For malnourished children at enrollment*: Incidence of WHZ < − 3, MUAC < 115 mm, nutritional oedema. Or > 10% weight loss during follow up. Powered for combined outcome of negative nutritional outcome^vii^	Low ^ϑ, Ψ^
Oral Rehydration Solution Formulations								
Santosham 1983 [39]	Panama	Inpatient 3 mo - 2 y/o who were well nourished, with acute diarrhea (more than 3 watery stools per day)	High potassium and chloride ORS, or standard WHO-ORS	Standard diet for diarrhea management (aerated beverages, bananas, cereals, and apple sauce)	93	14 days	Weight at day 14, weight gain at day 14 as percent of enrollment weight (power/sample size calculations not reported)	Very low^Ω, Φ, Ψ^
Ribeiro 1991 [40]	Brazil	Inpatient male infants less than 12 mo, with acute diarrhea and dehydration	Standard WHO-ORS with 30 mmol/L alanine	Standard WHO-ORS	18	7 days	Weight gain at day 7 (power/sample size calculations not reported)	Moderate^Ω^
Faruque 1997 [21]	Bangladesh	Inpatient children 3–35 mo with acute non-dysenteric diarrhea	Glucose based ORS	Rice-powder based ORS	471	16 days	Proportion with diarrhea at day 14, weight gain at day 16 (powered for outcomes of stool output, diarrhea duration and weight gain [70 g])	Low ^Φ, Ψ^
Alam 2009 [55]	Bangladesh	Severely malnourished (< 70% NCHS standard), inpatient infants 6–60 mo with acute diarrhea and culture-confirmed *V. cholerae*	Glucose-based ORS, or Glucose-based ORS plus amylase resistant starch	Rice-based ORS	137	6 weeks	Time to attain 80% of median WLZ from enrollment; proportion with diarrhea at or after day 7 (power/sample size calculations not reported)	Low^Ω, Ψ^
Probiotics								
Boudraa 2001 [47]	Algeria	Inpatient children 3–24 mo with acute watery diarrhea (> 3 loose stools in the previous 24 h)	Standard formula fermented with *L. bulgaricus* and *S. thermophilus* (lactose and calorically equivalent)	Standard milk-based formula	97	7 days	Weight gain at day 7 (power/sample size calculations not reported)	Very low^Ω, Φ, ϑ, Ψ^
Villaruel 2007 [41]	Argentina	Outpatient children 3 mo - 2 yo, with acute diarrhea (3 or more liquid or loose stools in the preceding 24 h)	WHO-ORS and S. boulardii, 250 mg per day (patients < 1 yo) or 500 mg per day (patients 1 yo and older)	WHO-ORS and placebo	72	1 month	Proportion of patients with diarrhea at or after day 7 (power/sample size calculations not reported)	Low^Ω, ϑ^
Misra 2009 [19]	India	Inpatient infants < 36 mo with diarrhea (more than 3 stools per day that take the shape of their container)	Lactobacillus rhamnosus GG (10^9 live bacteria)	Placebo	207	6 weeks	Change in WHZ at 6 weeks(powered for outcomes of stool output and diarrhea duration)	High
Sindhu 2014 [20]	India	Children 6 m to 5 years with diarrhea testing positive for either rotavirus or Cryptosporidium infection	Lactobacillus rhamnuosus GG (10^10 organisms)	Placebo	123	4 weeks	Proportions stunted (HAZ < − 2 SD), underweight (WAZ < − 2 SD), and wasted (WHZ < − 2 SD) at 4 weeks, proportion with diarrhea or severe diarrhea during follow-up (powered for outcome of L:M ratio)	Moderate^Ω^
Dinleyici 2014 [52]	Turkey	Inpatient children 3–60 mo with acute watery diarrhea	WHO-ORS + *lactobacillus reuteri* 17,938 (10^8 CFU) for 5 days	WHO-ORS only	127	12 days	Proportion with diarrhea at day 12 (powered for outcome of diarrhea duration)	Very low^Ω, Φ,Ψ^
Therapeutic Micronutrients (Vitamin A and Zinc)^viii^								
Faruque 1999 [34]	Bangladesh	Inpatient children 6 mo - 2 yo with acute diarrhea (3 or more liquid stools in the previous 24 h)	4500 μg vitamin A, 15 day, 14.2 mg Zinc acetate, 15 days, or both^ix^	Placebo	656	17 days	Proportion with diarrhea at day 7 and 16 (powered for outcome of diarrhea duration)	High
Khatun 2001 [22]	Bangladesh	Inpatient children 6 mo - 4 yo with persistent diarrhea (diarrhea for > 14 days duration)	Multivitamin (D, C, B1 B2 B6) syrup and 20 mg elemental zinc (as zinc acetate, 5 ml twice daily for 7 days), multivitamin syrup with Vit A (100,000 IU for children < 1 yo, 200,000 for children > 1 yo), or both	Multivitamin (D, C, B1 B2 B6) syrup only	93	7 days	Weight at day 7, weight gain at day 7 compared to day 1, proportions with diarrhea at day 7 (powered for outcome of clinical recovery)	Moderate^Ω^
Therapeutic Micronutrients (Zinc Alone)								
Sazawal 1995 [23]	India	Inpatient children 6–35 mo, with acute diarrhea (at least 4 unformed stools in the preceding 24 h)	Multivitamin syrup (A, B2, B6, D3, E) plus zinc gluconate (20 mg of elemental zinc)	Multivitamin syrup (A, B2, B6, D3, E) only	937	At least 120 days	Proportion of diarrhea episodes that last longer than 7 days, proportion of diarrhea episodes taken to a physician during follow up (power/sample size calculations not reported)	High
Roy 1998 [24]	Bangladesh	Inpatient 3–24 mo with persistent diarrhea	Multivitamin syrup (Vit A, B1, B2, B3, B6, D, Ca) with 20 mg elemental zinc per day for 14 days	Multivitamin syrup (Vit A, B1, B2, B3, B6, D, Ca) only for 14 days	141	15 days	Mortality, weight gain at discharge compared to admission weight, proportion with diarrhea after day 15 (powered for outcome of diarrhea duration)	Very low^Ω, Φ, ϑ^
Bhutta 1999 [49]	Pakistan	Inpatient children 6–36 mo with persistent diarrhea	Multivitamin syrup (Vit A, B1, B2, B3, B6, B12, C, D, Ca) with 3 mg elemental zinc per kg per day for 28 days	Multivitamin syrup (Vit A, B1, B2, B3, B6, B12, C, D, Ca) only for 28 days	77	28 days	Weight gain at day 7 and 14; overall weightincrement at day 14; MUAC at day 7 and 14; overall MUAC increment (powered for outcome of day 14 weight gain)	Low^Ω, ϑ^
Roy 1999 [25]	Bangladesh	Malnourished (< 76% of NCHS median), inpatient children 3–24 months with acute diarrhea	Multivitamin syrup (vit A, B1, B2, B6, D, and Ca) with 20 mg elemental zinc per day for 14 days	Multivitamin syrup (vit A, B1, B2, B6, D, and Ca) only for 14 days	29	10 weeks	Weight gain at each week of for 8 weeks, length gain at each week for 8 weeks (power/sample size calculations not reported)	Low^Ω, ϑ^
Baqui 2002 [27]	Bangladesh	Community-based children 3–59 mo with diarrhea of any duration	ORS with 20 mg zinc per day, 14 days	ORS only	8070	2 years^x^	Incidence of diarrhea, mortality (powered for the outcomes of diarrhea duration, diarrhea incidence, acute lower respiratory infections incidence, admission to hospital for diarrhoea or acute lower respiratory infections, and child mortality)	Low ^Φ, Ψ^
Walker 2007 [53]	Ethiopia, Pakistan, and India	Infants 1–5 mo with acute diarrhea, identified through home visits by health workers and community based study clinics	ORS with 10 mg zinc sulfate, daily for 14 days	ORS with placebo	1042	8 weeks	Weight at week 4 and 8, length at week 4 and 8, proportion of infants with ≥1 episode of any diarrhea, ≥ 2 episode of any diarrhea, or ≥1 episode of dysentery (any day with blood in the stool); incidence and prevalence of diarrhea; mortality (powered for anthropometry and morbidity outcomes)	High
Roy 2007 [28]	Bangladesh	Convalescent children 3–24 mo, after recovery from persistent diarrhea	Multivitamin syrup (Unspecified) with 20 mg elemental zinc, 14 days	Multivitamin syrup (unspecified) only, 14 days	147	12 weeks	Mortality, gain in length and weight at 12 weeks, incidence of subsequent diarrhea episodes (power/sample size calculations not reported)	Low^Ω, Φ, ϑ^
Roy 2008 [29]	Bangladesh	Moderately malnourished (weight/age 61–75% of NCHS median), inpatient children age 12–59 m with acute bloody-mucoid diarrhea or febrile diarrhea, and lab-confirmed *Shigella spp*	Multivitamin syrup (A, D, B complex, Ca) with zinc acete (10 mg elemental Zn/5 mL), for 14 days	Multivitamin syrup (Vit A, D, B complex, Ca) only	30^xi^	6 months	Diarrhea incidence and duration of episodes during 6 mo follow up (power/sample size calculations not reported)	Low^Ω, ϑ^
Fajolu 2008 [48]	Nigeria	Outpatient children 6–24 mo with acute diarrhea (3 or more loose, liquid or watery stools in a 24 h period)	20 mg of elemental zinc (zinc sulphate monohydrate) for patients > 1 y/o, 10 mg of elemental zinc, 14 days, for patients < 1 y/o	Placebo	60	3 months	Weight gain at 3 months, number and duration of subsequent diarrhea episodes during follow up (power/sample size calculations not reported)	Moderate^Ω^
Larson 2010 [30]	Bangladesh	Community-based children 6–23 mo with acute diarrhea and culture-confirmed ETEC	10 days of zinc (10 mg/d) + additional 3 months of zinc supplementation (10 mg/d)	10 days of zinc (10 mg/d) only	333	9 months	Incidence rate of diarrhea illness during follow up (powered for the incidence of acute upper respiratory tract infections)	Moderate^ϑ^
Alam 2011 [31]	Bangladesh	Community-based children 4–59 mo with diarrhea (3 or more loose or liquid stools in the previous 24 h)	Short course zinc - 20 mg elemental zinc, 5 days	Standard course zinc - 20 mg elemental zinc, 10 days	1622	90 days	Number of diarrheal episodes and days of diarrhea during follow up; proportion with at least 1 subsequent episode of diarrhea, prolonged diarrhea, or persistent diarrhea during follow up; day of onset of first subsequent diarrhea episode during follow up (powered for the outcome of diarrhea incidence)	High
Patel 2013 [32]	India	Outpatient children 6–59 mo with acute diarrhea (> 3 unformed stools in the previous 24 h per mother’s report)	Zinc (2 mg/kg/day) or zinc + copper (Zn 2 mg/kg/day + Cu 0.2 mg/kg/day), 14 days	Placebo	724	12 weeks	Proportion with at least 1 diarrhea episode, 2 diarrhea episodes, or 1 dysentery episode during follow up; number and duration of subsequent diarrhea episodes; change in WAZ, WHZ, and HAZ from enrollment measurements every 2 weeks for 12 weeks (power/sample size calculations not reported)	High
Negi 2015 [33]	India	Children 5–12 yrs. presenting to pediatric emergency units with acute watery diarrhea (3 or more episodes of loose stools over 24 h of < 72 h duration), with some or severe dehydration, and having had no treatment	Zinc (20 mg/day) for 14 days	Placebo	134	3 months	Risk of having at least 1 episode of diarrhea during follow up (power/sample size calculations not reported)	Low^Ω, ϑ^

^i^Outcome listed are only the outcomes of interest for the present systematic review

^ii^Duration of diets was variable. Diets were started at low concentrations and were advanced every 48 hours if no sign of intolerance. If there were signs of intolerance, diets were maintained or decreased as necessary. When full concentrations were reached, the diet was given for an additional 7 days.

^iii^Defined as when diarrhea had ceased and patient had consistent weight gain for at least 48 hours

^iv^Defined as age- and sex-specific weight standard deviation scores, from the National Center for Health Statistics median value

^v^All diets were equivalent in calorie and protein composition

^vi^Study included children with multiple admission (not just diarrhea) therefore only included data for children who had diarrhea at time of treatment. Power was determined for all children (not stratified by diagnosis)

^vii^Study included children with multiple admission (not just diarrhea) therefore only included data for children who had diarrhea at time of treatment. Power was determined for all children (not stratified by diagnosis)

^viii^Studies in this intervention category are randomized controlled trials with a factorial design, evaluating both Vitamin A and Zinc

^ix^Investigators included 2 strata of study subjects: A “standard dose stratum” with the dosages given, and a “High dose stratum” with 40 mg zinc acetate daily, 15 days (Vit A dosage was unchanged)

^x^Diarrhea morbidity data were collected from “samples of time periods” throughout the 2 year follow up period. Mortality rates were calculated using 11,881 child-years of person-time, and incidence rates were calculated using 41,788 child-weeks of person-time

^xi^Thirty completed 6 month follow up; 50 completed 7-day clinical study

^Ω^Sparse data (sample size is <200 participants total)

^Φ^Blinding and allocation process (not double-blind)

^ϑ^Follow up and withdrawals (>5% of sample size)

^Ψ^Lack of placebo

Of the 46 clinical trials evaluated using the modified GRADE system, 6 (13.0%) scored high, 8 (17.4%) scored moderate, 11 (23.9%) scored low, and 21 (45.7%) scored very low (Table 1). The most common deduction was for sparse data (35 [76.1%]), followed by deductions related to follow-up and withdrawals (24 [52.2%]), or blinding/allocation process (21 [45.7%]).

### Mortality

**Fig. 2:**
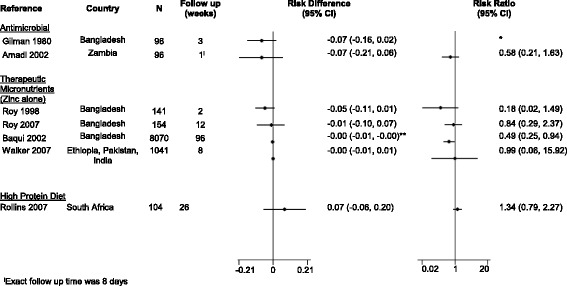
Effect of diarrhea management interventions on mortality (relative risk, risk difference, and associated 95% confidence interval)

Seven studies (15.2%) presented data on post-acute mortality, with follow-up ranging from 8-days to 2 years. Four were trials of therapeutic zinc interventions, [24, 27, 28, 53], 2 antimicrobial treatments [8, 42], and 1 of a high protein diet [44]. Two of the zinc trials were large studies (8070 and 1042 subjects, respectively) [27, 53] but the remaining 5 included less than 150 participants (Fig. 2, Table 2). None of the 7 trials were adequately powered for a mortality endpoint. Only 1 trial, a cluster randomized trial of zinc, found a lower non-injury mortality rate in children living in communities randomized to ORS and zinc compared to those using ORS alone (relative risk [RR] = 0.49 [95% confidence interval {95% CI}: 0.25, 0.94]) [27]. The remaining 6 studies reported non-significant risk differences ranging in magnitude from 70 more to 105 less deaths per 1000 children [8, 44] and relative risks ranging from 0.18 to 1.34 [24, 44].

**Table 2:** Intervention effects on post-acute (≥7 days after enrollment) mortality, length, weight, and/or diarrhea presence

Reference	Intervention	Comparator	Relevant Outcomes and Results		
Antimicrobial Interventions					
Gilman 1980 [8]	Low dose ampicillin (50 mg/kg/day)	Standard dose ampicillin (150 mg/kg/day)	Mortality	Risk Difference (95% CI)	−0.11 (− 0.24, 0.03)^i^
				Relative Risk (95% CI)	Undefined
Amadi 2002 [42]	5 mL of 20 g/L nitazoxanide, twice daily for 3 days	Placebo	Mortality	Risk Difference (95% CI)	−0.07 (− 0.21, 0.06)
				Relative Risk (95% CI)	0.58 (0.21, 1.63)
Dietary Supplements					
Alam 2000 [26]	ORS with dietary fiber (Benefiber®)	WHO- ORS only	Difference in weight gain at day 7, g (95% CI)	52 (−18.73, 122.73)	
Rabbani 2001 [15]	Rice-based diet with dietary fiber (green banana or pectin supplement), 7 days	Rice-based diet only	Proportions recovered from diarrhea (formed stool) at each day to day 10	Higher in banana and pectin groups than in control group^ii^	
Yalcin 2004 [51]	Glutamine supplement, 0.3 g/kg/day, for 7 days	Placebo	Difference in weight gain at day 30, g (95% CI)^iii^	130 (12.67, 247.33)	
			Difference in weight gain at day 60, g (95% CI)	45 (−80.80, 170.80)	
			Difference in weight gain at day 90, g (95% CI)	107 (−57.30, 271.30)	
High Protein Diets					
Datta 1990 [9]	High protein diet, length unspecified	Standard hospital diet	Difference in weight at day 15, kg (95% CI)	0.30 (−0.18, 0.78)	
			Difference in MUAC at day 15, cm (95% CI)	0.00 (−0.37, 0.37)	
Kabir 1992 [10]	High protein diet (15% of total energy from protein), 21 days	Standard diet (7.5% of total energy from protein), 21 days	Difference in change in weight at day 21 from admission, kg (95% CI)	0.47 (0.12, 0.82)	
			Difference in change in height at day 21, from admission, cm (95% CI)	0.09 (−0.57, 0.75)	
			Difference in change in WAZ at day 21 from admission (95% CI)	0.30 (0.03, 0.57)	
			Difference in change in WHZ at day 21 from admission (95% CI)	0.40 (0.05, 0.75)	
			Difference in change in MUAC at day 21 from admission, cm (95% CI)	0.44 (0.08, 0.80)	
			Difference in change in triceps skinfold thickness at day 21 from admission, cm (95% CI)	0.32 (−0.29, 0.93)	
Kabir 1993 [11]	High protein diet, 21 days	Standard protein diet, 21 days	Difference in change in WAZ at day 21, from admission (95% CI)	0.23 (0.07, 0.39)	
			Difference in change in WHZ at day 21, from admission (95% CI)	0.25 (0.05, 0.45)	
			Difference in change in HAZ at day 21, from admission (95% CI)	0.90 (0.05, 0.13)	
Mazumder 1997 [12]	High calorie & high protein milk-cereal diet, 4960 kJ/l (10 days)	Standard diet, 2480 kJ/l (10 days)	Difference in percent change in WAZ at day 10 and 40, fromadmission (95% CI)	Day 10: 3.50 (1.86, 5.14)	
				Day 40: 3.11 (0.92, 5.30)	
			Difference in percent change in WHZ at day 10 and 40, from admission (95% CI)	Day 10: 3.76 (1.92, 3.60)	
				Day 40: 3.34 (0.76, 5.90)	
Nurko 1997 [36]	High protein chicken-based diet or high protein soy-based diet (Nursoy formula)^iv^	Calorically equivalent standard “elemental diet” (Vivonex)	Difference in weight at end of protocol, g^v^ (95% CI)	Comparing soy group to control	− 204 (− 1178.40, 770.40)
				Comparing chicken group to control	−445 (− 1522.70, 632.70)
			Difference in weight at discharge from enrollment, g (95% CI)	Comparing soy group to control	−92 (− 1189.60, 1005.60)
				Comparing chicken group to control	− 428 (− 1539.80, 683.80)
			Proportion with nutritional recovery^vi^(RR [95%])	Comparing soy group to control	1.13 (0.79, 1.61)
				Comparing chicken group to control	1.20 (0.86, 1.7)
Kabir 1998 [13]	High protein diet (15% of total energy from protein), 21 days	Standard protein diet (7.5% of total energy from protein), 21 days	Difference in change in weight at 6 mo, from post-intervention weight, kg (95% CI)	0.10 (−0.24, 0.44)	
			Difference in change in height at 6 mo, from post-intervention height, cm (95% CI)	1.10 (0.56, 1.64)	
			Difference in change in WAZ at 6 mo, from post-intervention WAZ (95% CI)	0.07 (−0.17, 0.31)	
			Difference in change in WHZ at 6 mo, from post-intervention WHZ (95% CI)	−0.09 (− 0.35, 0.17)	
			Difference in change in HAZ at 6 mo, from post-intervention HAZ (95% CI)	0.28 (0.12, 0.44)	
Mazumder 2000 [14]	High calorie & protein milk-cereal formula (4960 kJ/l), 10 days	Control milk-cereal formula (2480 kJ/l), 10 days	Difference in percent change in WAZ at day 10, from admission WAZ (95% CI)	3.50 (2.08, 4.91)	
Valentiner-Branth 2001 [43]	Counseling on the importance of breastfeeding and of a nutritious diet, and a high protein millet gruel with a multivitamin tablet (including zinc), until the end of a 7 day period without diarrhea	Counseling on the importance of breastfeeding and of a nutritious diet	Difference in weight gain at end of intervention and day 90, g/wk.^vii^(95% CI)	Day 90	61.50 (49.20, 73.80)
				End of intervention	12.50 (7.70, 17.30)
			Difference in change in knee heel length at end of intervention and day 90, mm/y (95% CI)	Day 90	2.70 (−4.60, 10.00)
				End of intervention	7.50 (4.80, 10.20)
			Difference in change in height between groups at day 90, (cm/y)^2^ (95% CI)^viii^	0.65 (0.11, 1.19)	
Rollins 2007 [44]	Standard nutritional support + extra protein to provide 150 kcal/kg/day and 4.0–5.5 g protein/kg/day	Standard nutritional support: maize porridge + milk formula	Mortality at 26 weeks	Relative risk	1.34 (95% CI: 0.79, 2.27)
				Risk difference	7.4% (−4.7%,20.5%)
			Median change in weight- SDs ^ix^at 26 weeks	Greater gain in intervention group (*p* < 0.001)	
			Median change in WAZ at 26 weeks	Greater gain in intervention group (*p* < 0.05)	
			Proportion underweight (WAZ < −2 SDs) at 26 weeks (Prevalence Ratio [95% CI])	0.48 (0.30, 0.77)	
			Proportion stunted (LAZ < − 2 SDs) at 26 weeks (Prevalence Ratio[95% CI])	0.87 (0.67, 1.13)	
Lactose Free Diets					
Bhan 1988 [16]	Legume and cereal-based formula (lactose-free), until recovery or 7 days min	Calorically equivalent milk-based formula, until recovery or 7 days min	Difference in weight gain at day 7, g/kg admission weight/24 h (95% CI)	−3.20 (−6.86, 0.46)	
			Difference in weight gain at recovery, g/kg admission weight/24 h (95% CI)	−3.80 (−7.15, −0.44)	
Bhutta 1991 [50]	Soy milk (lactose-free) for 7 days, followed by khitchri and yogurt for 7 days	Khitchri and yogurt for 14 days	Difference in weight gain at day 7 and 14, g/wk. (95% CI)	Day 7: −400 (− 559.40, − 240.60)	
				Day 14: 385.7 (209.60, 561.80)	
Lozano 1994 [38]	Corn-based (lactose-free) formula, 21 days	Milk-based formula, 21 days	Difference in weight increment at 6 weeks, kg (95% CI)	−0.02 (− 0.30, 0.26)	
Bhatnagar 1996 [17]	Rice-based formula with egg white protein (lactose-free), until discharge	Rice-based formula with milk protein, until discharge	Proportion of patients whose weight on day 7 was lower than at rehydration (Prevalence Ratio [95% CI])	0.97 (0.06, 15.19)	
			Probability of continuing diarrhea at each day to day 12	No significant difference (p = 0.76)^x^	
de Mattos 2009 [37]	Amino-acid based diet, isolated soy-based, or hyrolyzed casein-based diet; until discharge	Yogurt-based diet^xi^ until discharge	Difference in weight gain at discharge among groups at discharge	No difference among groups^xii^	
			Change in WHZ at discharge	Similar improvement in all groups^xiii^	
Other Dietary Interventions					
Eichenberger 1984 [35]	Semi-elemental diet with low osmolarity and high hydrolyzed lactalbumin, at least 21 days	Standard hospital diet	Weight at day 21 compared to weight at beginning of therapy	Better in intervention group^xiv^	
van der Kam 2016 (Uganda) [45]	RUTF, plus instructions to feed the child an extra meal/day for 14 d; or micronutrient powder plus instructions to feed the child an extra meal/day for 14 d	An instruction to feed the child an extra meal/day for 14 d	Relative Risk of first event of malnutrition (95% CI)	RUTF vs micronutrient group	0.68 (0.37, 1.22)
				RUTF vs control group	0.62 (0.35, 1.10)
				Micronutrient group vs control	0.92 (0.54, 1.54)
van der Kam 2016 (Nigeria) [46]	RUTF, plus instructions to feed the child an extra meal/day for 14 d; or micronutrient powder plus instructions to feed the child an extra meal/day for 14 d	An instruction to feed the child an extra meal/day for 14 d	Relative Risk of first event of malnutrition (95% CI)	RUTF vs micronutrient group	1.12 (0.84, 1.50)
				RUTF vs control group	0.91 (0.69, 1.20)
				Micronutrient group vs control	0.81 (0.61,1.09^xv^)
ORS Formulations					
Santosham 1983 [39]	High potassium/ high chloride ORS, or standard WHO-ORS, with regular diet	Standard diet for diarrhea management	Difference in weight at day 14, kg (95% CI)	Comparing high potassium/chloride to control	−0.3 (−1.45, 0.85)
				Comparing standard ORS to control	−0.50 (− 1.86, 0.76)
			Difference in percent weight gain at day 14 (95% CI)	Comparing high potassium/chloride to control	2.00 (1.57, 2.42)
				Comparing standard ORS to control	2.30 (1.82, 2.77)
Ribeiro 1991 [40]	Alanine-based ORS	Standard WHO-ORS	Difference in weight gain at day 7, g (95% CI)	18 (−94.37, 130.38)	
Faruque 1997 [21]	Glucose-based ORS	Rice powder-based ORS, equivalent in electrolyte content	Proportion with diarrhea at day 14 (RR [95% CI])	0.80 (0.21, 2.95)	
			Difference in weight gain at day 16, g	Similar between groups^xvi^	
Alam 2009 [55]	Glucose-based ORS, or glucose-based ORS with amylase-resistant starch (ARS)	Rice-based ORS	Difference in time to attain 80% of median WLZ, days (95% CI)	Difference between glucose and rice ORS	− 0.06 (− 1.19, 1.07)
				Difference between glucose with amylase resistant starch and rice ORS	− 0.08 (− 1.20, 1.04)
			Proportion with diarrhea at or after day 7 (RR [95% CI])	Risk in glucose ORS group compared to rice ORS group (RR, [95% CI])	1.00 (0.15, 6.86)
				Risk in glucose with ARS group compared to rice ORS groups (RR, [95% CI])	0.49 (0.05, 5.27)
Probiotics					
Boudraa 2001 [47]	Milk-based formula fermented with *L. bulgaricu*s *S. andthermophilus*	Milk-based formula only	Difference in weight gain at day 7, g (95% CI)	43 (− 109.18, 195.18)	
			Difference in weight gain at day 7, g/kg (95% CI)	4.4 (−5.50, 14.30)	
Villarruel 2007 [41]	*S. boulardii* capsules	Placebo	Proportion with diarrhea on day 7 (Prevalence Ratio [95% CI])	0.39 (0.20, 0.74)	
			Proportion with diarrhea after day 7 (Prevalence Ratio [95% CI])	0.25 (0.07, 0.82)	
Misra 2009 [19]	*L. rhamnosus* GG 10^9^ CFU	Placebo	Difference in change in WHZ at 6 weeks	No difference between groups (*p* = 0.06)	
Sindhu 2014 [20]	*L. rhamnosus* GG 10^10^ CFU	Placebo	Proportion with diarrhea at 4 weeks follow up (Prevalence Ratio [95% CI])	0.65 (0.40, 1.07)	
			Proportion with severe diarrhea during follow-up (Prevalence Ratio [95% CI])	1.15 (0.65, 2.05)	
			Proportion with diarrhea requiring hospitalization during follow-up (Prevalence Ratio [95% CI])	1.03 (0.54, 1.98)	
			Proportion stunted (HAZ < −2 SD) at week 4 (Prevalence Ratio [95% CI])	1.77 (1.00, 3.13)	
			Proportion wasted (WHZ < − 2 SD) at week 4 (Prevalence Ratio [95% CI])	0.53 (0.16, 1.71)	
			Proportion underweight (WAZ < − 2 SD) at week 4 (Prevalence Ratio [95% CI])	1.66 (0.83, 3.30)	
Dinleyici 2014 [52]	ORS with *L. reuteri* 17,938 10^8^ CFU	ORS only	Proportion with diarrhea at day 12 (Prevalence Difference [95% CI])^xvii^	0.17 (0.08, 0.26)	
Therapeutic Micronutrients (Vitamin A and Zinc)					
Faruque 1999 [34]	Vitamin A (4500 μg retinol equivalent daily for 15 days), zinc acetate (14.2 mg daily for 15 days),^xviii^or both^xix^	Placebo	Proportion with diarrhea on day 7 (Prevalence Ratio [95% CI])	Zinc-supplemented vs. non-supplemented	0.64 (0.43, 0.96)
				Vitamin A –supplemented vs. non-supplemented	0.78 (0.52, 1.49)
			Proportion with diarrhea on day 16 (Prevalence Ratio [95% CI])	Zinc-supplemented vs. non-supplemented	0.67 (0.24, 1.85)
				Vitamin A –supplemented group vs. non-supplemented	0.67 (0.24, 1.85)
Khatun 2001 [22]	Multivitamin syrup with 20 mg elemental zinc (twice daily for 7 days), or multivitamin syrup with Vitamin A^xx^ or both	Multivitamin syrup only	Difference in change in weight at day 7 compared to day 1, g^xxi^	Zinc group vs control	0.11 kg (*p* = 0.045)
				Vitamin A group vs control	0.07 kg (*p* = 0.21)
				Zinc+Vitamin A vs. control	0.06 kg (*p* = 0.074)
			Proportion with diarrhea at day 7, (Prevalence Ratio [95% CI])	Zinc group vs. control	0.23 (0.08, 0.71)
				Vitamin A group vs. control	0.92 (0.54, 1.59)
				Zinc+Vitamin A vs. control	0.62 (0.31, 1.21)
Therapeutic Micronutrients (Zinc alone)					
Sazawal 1995 [23]	Multivitamin syrup with 20 mg elemental zinc^xxii^	Multivitamin syrup only	Proportion of episodes lasting longer than 7 days (Prevalence Ratio [95% CI])	0.87 (0.65, 1.16)	
			Proportion of diarrhea episodes taken to a physician during follow up (Prevalence Ratio [95% CI])	0.78 (0.57, 1.07)	
Roy 1998 [24]	Multivitamin syrup with 20 mg elemental zinc, 14 days	Multivitamin syrup only	Mortality	Relative Risk (95% CI)	0.18 (0.02, 1.49)
				Risk Difference (95% CI)	−0.05 (− 0.11, 0.01)
			Change in weight at discharge, g^xxiii^	Mean body weight in intervention group was maintained while body weight decreased in control group	
			Proportion with diarrhea after day 15 (RR [95% CI])^xxiv^	0.99 (0.53, 1.88)	
Bhutta 1999 [49]	Multivitamin with 3 mg of elemental zinc per kg of body weight, 28 days	Multivitamin only	Difference in weight at day 7 and 14, kg^xxv^ (95% CI)	Day 7: −0.57 (−1.14, − 0.002)	
				Day 14: −0.46 (− 1.06, 0.14)	
			Difference in overall weight increment at day 14, g/kg/day	1.60 (− 1.48, 4.68)	
			Difference in MUAC at day 7 and 14, cm (95% CI)	Day 7: −0.30 (− 0.98, 0.38)	
				Day 14: −0.40 (−1.08, 0.28)	
			Difference in overall MUAC increment, cm (95% CI)	0.00 (−0.13, 0.13)	
Roy 1999 [25]	Multivitamin with 20 mg of elemental zinc, 14 days	Multivitamin only	Difference in weight gain at each week of 8 week follow up, g (95% CI)	Week 1: 30 (−204.70, 264.73)	
				Week 2: −4 (− 202.60, 194.60)	
				Week 3: −45 (−303.70, 213.70)	
				Week 4: −60 (− 347.70, 227.70)	
				Week 5: −79 (−361.40, 203.40)	
				Week 6: −57 (− 354.38, 240.40)	
				Week 7: −53 (− 352.70, 246.70)	
				Week 8: −19.00 (− 394.15, 356.15)	
			Difference in gain in length at week 8, mm	4.40 mm, 30% greater gain (*p* < 0.03)^xxvi^	
Baqui 2002 [27]	ORS with 20 mg zinc per day, 14 days	ORS only	Incidence of diarrhea during 2 year follow up	RR (95% CI)	0.85 (0.76, 0.96)
				RD (95% CI)^xxvii^	2.9 (0.80, 5.10)^xxviii^
			Mortality during 2 year follow up	RR (95% CI)	0.49 (0.25, 0.94)
				RD (95% CI)	2.2 (0.60, 3.70)^xxix^
Walker 2007 [53]	ORS with 10 mg zinc, 14 days	ORS with placebo	Difference in weight at week 4 and 8, kg (95% CI)	Week 4: 0.06 (−0.08, 0.20)	
				Week 8: 0.06 (− 0.08, 0.20)	
			Difference in length at week 4 and 8, cm (95% CI)	Week 4: −0.09 (− 0.61, 0.43)	
				Week 8: −0.12 (− 0.63, 0.39)	
			Proportion of infants with ≥1 episode of any diarrhea (RR [95% CI])^xxx^	1.01 (0.92, 1.12)	
			Proportion of infants with ≥2 episode of any diarrhea (RR [95% CI])	0.79 (0.67, 0.95)^xxxi^	
			Proportion of infants with ≥1 episode of dysentery (any day with blood in the stool) (RR [95% CI])	2.10 (0.96, 4.61)	
			Incidence of diarrhea (episodes/month)	Intervention group (mean ± SD)	0.62 ± 0.68
				Control group (mean ± SD)	0.61 ± 0.70
			Prevalence of diarrhea (days/mo)	Intervention group (mean ± SD)	2.68 ± 4.11
				Control group (mean ± SD)	2.20 ± 3.19
			Mortality	RR (95% CI)	0.99 (0.01, 77.9)
				RD (95% CI)^xxxii^	−0.18 (−66.42, 66.06)
Roy 2007 [28]	Multivitamin with 10 mg zinc per 5 ml, 14 days	Multivitamin only	Difference in mean number of diarrhea episodes during 6 mo follow up (95% CI)^xxxiii^	− 0.33 (− 0.39, − 0.27)	
			Percent gain in length at 12 weeks, mm^xxxiv^	Comparable between groups when all patient were compared (*p* = 0.6)	
				24% greater among underweight (WAZ ≤ 70% NCHS median) (p < 0.03)	
			Mortality	RR (95% CI)	0.84 (0.29,2.37)
				RD (95% CI)	−0.01 (−0.10, 0.07)
Roy 2008 [29]	Multivitamin with 20 mg zinc, 14 days	Multivitamin only	Geometric mean diarrhea incidence	Statistically significantly higher in control group (*p* = 0.03)	
Fajolu 2008 [48]	20 mg zinc for patients > 1 y; 10 mg zinc for patients < 1 y	Placebo	Number of subsequent diarrhea episodes during 2 month follow up	Difference not significant (*p* = 0.53)	
			Weight gain at 2 months, g	Higher in intervention group (*p* < 0.001)	
Larson 2010 [30]	10 days therapeutic zinc (20 mg) followed by 3 mo supplementary zinc (10 mg)	10 days therapeutic zinc (20 mg) followed by 3 mo supplementary zinc placebo	Diarrhea episodes during follow up,^xxxv^ months 1–3 Risk Difference, [95% CI])	−1.02 (0.26, 1.79)	
			Diarrhea episodes during follow up, months 4–6 (Risk Difference, [95% CI])	−0.37 (− 0.35, 1.07)	
			Diarrhea episodes during follow up, months 7–9 (Risk Difference, [95% CI])	−0.18 (− 0.41, 0.75)	
			Diarrhea episodes during entire 9 month follow up (Risk Difference, [95% CI])	−0.54 (0.07, 1.01)	
Alam 2011 [31]	Short course zinc – 20 mg zinc, 5 days	Standard course zinc – 20 mg zinc, 10 days	Difference in mean number of diarrhea episodes during 3 month follow up (95% CI)	0.06 (− 0.07, 0.19)	
			Difference in mean number of days of diarrhea during 3 month follow up (95% CI)	0.2 (−0.35, 0.75)	
			Proportion of children with at least 1 episode of diarrhea during 3 month follow up (RR [95% CI])	1.03 (0.95, 1.14)	
			Proportion of children with prolonged diarrhea (≥ 7 days) (Prevalence Ratio [95% CI])^xxxvi^	0.63 (0.50, 0.79)	
			Proportion of children with persistent diarrhea (≥ 14 days) (Prevalence Ratio [95% CI])	0.63 (0.48, 0.81)	
			Day of onset of first subsequent diarrhea episode during follow up	No difference between groups^xxxvii^	
Patel 2013 [32]	Zinc (2 mg/kg/day) or zinc + copper (Zn 2 mg/kg/day + Cu 0.2 mg/kg/day), 14 days	Placebo	Proportion with at least 1 diarrhea episode during 3 month follow up (Prevalence Ratio [95% CI])	Zinc group vs placebo	1.01 (0.76, 1.33)
				Zinc + Copper group vs placebo	0.96 (0.72, 1.27)
			Proportion with at least 2 episodes of diarrhea during 3 month follow up^xxxviii^ (Prevalence Ratio [95% CI])	Zinc group vs placebo	2.25 (1.10, 4.63)
				Zinc group vs Zinc + Copper group	1.12 (0.64, 1.97)
			Proportion with at least 1 dysentery episode during 3 month follow up (Prevalence Ratio[95% CI])	Zinc group vs placebo	1.31 (0.30, 5.77)
				Zinc group vs Zinc + Copper group	1.37 (0.31, 6.04)
			Difference in mean number of subsequent diarrhea episodes per child during 3 month follow up (95% CI)	Zinc group vs placebo	0.08 (−0.05, 0.21)
				Zinc + copper group vs placebo	0.05 (−0.07, 0.17)
			Difference in change in WAZ at month 3 (95% CI)	Zinc group vs placebo	−0.1 (− 0.12, 0.10)
				Zinc + copper group vs placebo	0.06 (−0.04, 0.16)
			Difference in change in WHZ at month 3 (95% CI)	Zinc group vs placebo	−0.01 (− 0.18, 0.16)
				Zinc + copper group vs placebo	0.09 (−0.07, 0.25)
			Difference in change in HAZ at month 3 (95% CI)	Zinc group vs placebo	0.02 (−0.09, 0.13)
				Zinc + copper group vs placebo	0.03 (−0.08, 0.14)
Negi 2015 [33]	Zinc (20 mg/day) for 14 days	Placebo	Risk of having at least 1 episode of diarrhea during 3 mo follow up (Relative risk [95%])	Among all subjects	0.65 (0.37, 1.23)
				Among zinc-deficient subjects (*n* = 60)	0.65 (0.31, 1.38)

^i^Estimate may be interpreted as 11 fewer deaths per 100 children in the intervention group compared to the control group

^ii^Quantitative estimates not presented and reported p-values not specific to time-points of interest

^iii^Data presented were assumed to be mean ± SD

^iv^Duration of diets was variable. Diets were started at low concentrations and were advanced every 48 hours if no sign of intolerance. If there were signs of intolerance, diets were maintained or decreased as necessary. When full concentrations were reached, the diet was given for an additional 7 days.

^v^Appropriate data for calculation of weight gain or difference in weight gain not presented

^vi^Defined as when diarrhea had ceased and patient had consistent weight gain for at least 48 hours

^vii^All outcome measurements were compared against measurements at entry, when the child had had diarrhea for 14 days

^viii^Units were assumed to be cm/y due to description of results in the manuscript (rather than (cm/y)2 as presented in the study’s Four)

^ix^Defined as age- and sex-specific weight standard deviation scores, from the National Center for Health Statistics median value

^x^No quantitative estimates given

^xi^All diets were equivalent in caloric and protein content

^xii^No estimate or statistical significance given

^xiii^No estimate or statistical significance given

^xiv^No estimates or statistical significance is given

^xv^Confidence interval states in manuscript is (0.605, 0.090) which does not contain the relative risk estimate of 0.812 therefore have assumed the 0.090 was a typo and replaced with 1.090.

^xvi^No estimates or statistical significance given

^xvii^RR was undefined due to a “0” cell

^xviii^The authors changed the dose after 417 children were enrolled (dosages listed, analyzed as "standard strata" of subjects), and the remaining 273 children received a higher dose of zinc (analyzed as "high dose strata"): Vitamin A (4500 ug retinol equivalent daily for 15 days) and/or zinc acetate (40 mg daily for 15 days)

^xix^Results from comparison of zinc+vitamin A vs. placebo not reported (reported on zinc effect by combing the zinc alone and zinc+vitamin A group and reported on vitamin A effect by combining vitamin A alone with the zinc+vitamin A group).

^xx^Vitamin A dosage was 100000 IU for children < 1 yo, and 200000 for children > 1 yo

^xxi^No SDs or CIs given for differences. P-value for the difference between zinc group and control is 0.045; for the difference between vitamin A group and control is 0.207; and for the difference between zinc + vitamin A group is 0.074.

^xxii^Duration of intervention is unclear

^xxiii^Data were presented but were not interpretable due to an ambiguous or incorrect title

^xxiv^Assessed by proportion of patients with delayed recovery, with recovery defined as the passage of formed stool followed by 2 days without diarrhea

^xxv^For this and all outcomes, data were not labeled. Data presented were assumed to be mean and SD based on labeled data on another figure in the paper

^xxvi^No SDs or CIs given. P< 0.05.

^xxvii^Difference in mean diarrhea incidence rates. Estimate may be interpreted as 2.9 more episodes per 100 child-years of observation were experienced in the control group compared to the intervention group

^xxviii^Calculated values of lower and upper limits of 95% CI differed from what was represented in original publication

^xxix^Difference in mean mortality rates. Estimate may be interpreted as 2.2 more deaths per 1000 child years of observation experienced by the control group compared to the intervention group

^xxx^All data presented are unadjusted. The authors’ results are discrepant from this table’s results, as authors adjust for original diarrhea episode lasting > 7 days, exclusive breastfeeding upon enrollment, and WLZ at beginning of follow up

^xxxi^Calculated values of lower and upper limits of 95% CI use data presented on unadjusted proportions and differed from what was represented in original publication

^xxxii^Estimate may be interpreted as 0.18 deaths fewer in intervention group per 100,000 child-weeks of observation compared to control group

^xxxiii^Data presented was assumed to be mean ± SD

^xxxiv^Quantitative estimates not presented

^xxxv^Risk differences may be interpreted as excess number of acute diarrhea episodes per child-year attributed to lack of zinc supplementation

^xxxvi^The authors reported "The proportion of prolonged (>=7 d) and persistent diarrhea episodes (>=14 d) did not vary between the 5-d (19 vs. 16%; P 0.08) and 10-d (12 vs. 10%; P = 0.14) groups" which suggests the p-values correspond to the comparison of persistent and prolonged among children treated with 5-days and among children treated with 10-days. We have instead assumed the appropriate comparisons are proportion of prolonged (19% vs. 12%, p-value=0.0001) and persistent (16% vs. 10%, p-value=0.0004) which would result in statistically significant differences (unlike what was reported).

^xxxvii^No estimates or statistical significance given

^xxxviii^Estimates calculated for relative risk of at least 1, 2, or dysenteric diarrhea episodes are discrepant from published results

### Growth

Studies reported impact on growth in several different ways:

- *Height/Length, HAZ//LAZ*

**Fig. 3:**
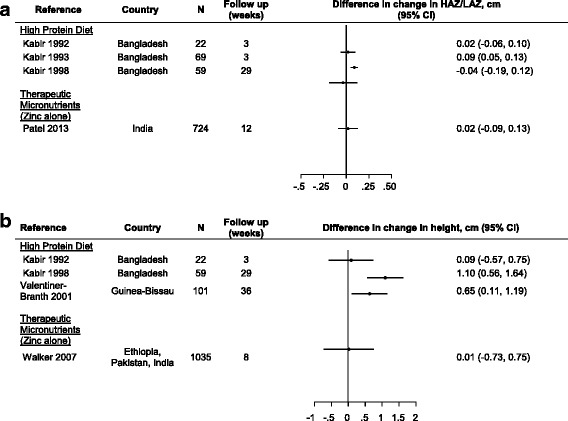
**a** Effect of diarrhea management interventions on change in HAZ/LAZ (difference in change in HAZ/LAZ and 95% confidence interval). **b** Effect of diarrhea management interventions on change in height (difference in change in height (cm) and 95% confidence interval)

Ten studies presented data related to length or height, with follow-up time ranging from 21 days to 9 months and none reported sample size calculations/being powered for these outcomes (Table 1). Five trials evaluated a high protein diet, 4 therapeutic zinc and 1 probiotic. Of 4 studies that reported difference in change in (∆) HAZ/LAZ between intervention groups, 1 high protein diet trial reported a 0.9 z-score greater gain in HAZ/LAZ in the intervention group after 3 weeks of follow-up (95% CI: 0.05, 0.13), [11] but 2 high protein diet trials (with 3 and 29 weeks of follow-up) and a large zinc with 12 weeks of follow-up trial found no significant benefit (Fig. 3a) [10, 13, 32]. Four studies presented data on ∆ absolute height at follow-up, 2 of which were trials of high protein diets with follow-up times of 3 and 29 weeks. Of the 2 high protein diet trials, the trial with 29 weeks of follow-up found a benefit in height change (Fig. 3b) – a 1.10 cm greater change in height compared to the control groups (95% CI: 0.56, 1.64) [10, 13]. Of the other 2 studies evaluating height attainment, a trial of a high protein diet with micronutrients with 36 weeks of follow-up, and another of therapeutic zinc with 8 weeks of follow-up, only the former showed benefit (a greater gain in height of 0.65 cm in the intervention group [95% CI: 0.11, 1.19]) [43, 53]. Percent ∆length was evaluated in 2 therapeutic zinc studies, both of which found a significantly greater length gain among children treated with zinc, but this result was only among underweight children in 1 of the trials [25, 28]. Among 2 studies evaluating prevalence of stunting during follow-up, 1 found that the group treated with probiotic*s (L. rhamnosus* GG) had higher stunting prevalence at 4 weeks of follow-up [54] and a high protein diet trial reported no significant difference at 26 weeks [44].

- *MUAC*

Four studies reported MUAC data during follow-up periods ranging from 15 to 28 days, 2 were high protein studies, 1 was a trial of therapeutic zinc, and 2 trials of RUTF/micronutrient (which assessed MUAC as 1 of the indicators of acute malnutrition). One of the high protein diet studies reported that children in the intervention group gained 0.44 cm more in MUAC on average compared to children given a standard protein diet (95% CI: 0.08, 0.80) [10]. However, neither the remaining high protein diet study nor the zinc trial reported a significant difference in MUAC during follow-up [9, 50]. None of the studies provided sample size estimates making it unclear whether they were adequately powered to detect differences in MUAC. Both trials of RUTF and micronutrient powder reported the incidence of acute malnutrition (WHZ < 2, MUAC < 115 mm, or oedema) to be similar across all combinations of groups (RUTF vs controls; micronutrients vs. controls, and RUTF vs. micronutrients) in the subgroup of children from both trials who had diarrhea at enrollment [45, 46].

- *WHZ, WAZ, or absolute weight*

**Fig. 4:**
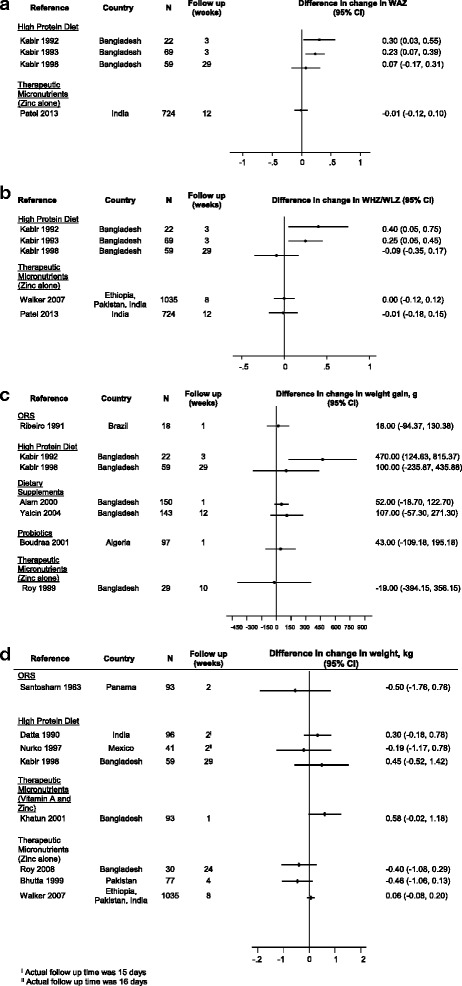
**a** Effect of diarrhea management interventions on change in WAZ (difference in change in WAZ and 95% confidence interval). **b** Effect of diarrhea management interventions on change in WHZ/WLZ (difference in change in WHZ/WLZ and 95% confidence interval). **c** Effect of diarrhea management interventions on weight gain (difference in weight gain [g] and 95% confidence interval). **d** Effect of diarrhea management interventions on weight at follow up (difference in weight [kg] and 95% confidence interval)

Thirty-two trials (74.4%) with follow-up periods ranging from 7 days to 29 weeks, reported data on weight, WAZ, or WHZ. Of these, 9 assessed a high protein diet, 7 assessed therapeutic zinc (including 1 which also assessed vitamin A), and 5 tested lactose-free diets. Four were trials of ORS formulations, 3 of probiotics, and 1 each of semi-elemental diet, glutamine, and dietary fiber. Of the 4 trials evaluating differences in ∆WAZ between study groups, 3 high protein and 1 therapeutic zinc trials, 2 (both high protein) reported a statistically significant improvement (ranging from 0.23 [11] to 0.3 z-scores [10]) compared to a standard diet (Fig. 4a) although none were explicitly powered for this outcome. The same 2 diet trials also reported a significant benefit in WHZ, with high protein groups gaining 0.25 [31] to 0.4 units [11] more in WHZ than the standard diet group (Fig. 4b) whereas the 2 zinc trials assessing ∆WHZ, 1 of which was explicitly powered to address WHZ, found no difference [32, 53]. Two additional trials assessed WHZ although did not present quantitative results for calculation of effect size and 95% confidence intervals; A probiotics trial concluded there was no difference in ∆WHZ at 6 weeks between the treated and untreated groups, [19] while a high protein diet trial reported a greater median ∆WAZ in children given high protein diets at 26 weeks of follow-up [44].

Twenty-two studies presented data on absolute weight gain (Fig. 4c) or weight at follow-up: 6 high protein diet trials, 6 zinc (1 of which also assessed vitamin A), 4 lactose free diets, 2 ORS, and 1 each of a probiotic, semi-elemental diet, dietary fiber, and glutamine. Three of the 6 high protein trials found a statistically significant improvement in weight associated with the intervention group, [10, 43, 44] as did 3 of the 6 zinc trials, [22, 25, 48] 1 of which also assessed vitamin A which did not appear to have a weight benefit [22]. Two of the 4 lactose-free diets [16, 50] and 1 of 2 ORS trials demonstrated a significant benefit in weight [39]. This trial found a greater percent improvement in weight 14 days after presentation in the groups of children treated with ORS (90 mmol/l or 50 mmol/l of sodium) vs. no ORS but did not find a statistically significant difference when measured as absolute difference in weight. Weight gain was significantly improved in the trial of a semi-elemental diet [35] and the single trial of glutamine found intervention children to have 130 g more weight gain than the placebo group at follow-up day 30, but not at days 60 or 90 of follow-up [51]. The single dietary fiber and probiotic trials evaluating weight gain did not find a significant effect [26, 47].

### Recurrent or prolonged diarrhea at follow-up

**Fig. 5:**
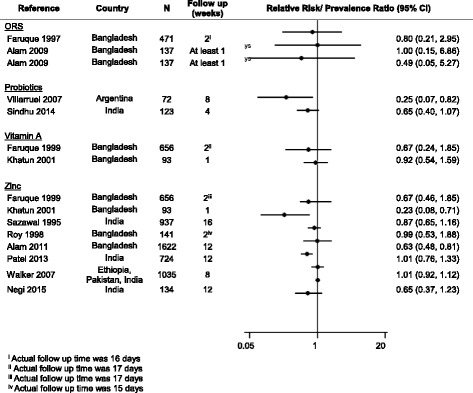
Effect of diarrhea management interventions on diarrhea morbidity during follow up (relative risk or prevalence ratio of diarrhea at specified time during follow up [95% CI])

Twenty studies (45.7% of total) reported on diarrhea frequencies during follow-up periods ranging from 7 days to 3 months. The majority were trials of therapeutic zinc (13), including 2 that also assessed vitamin A, followed by probiotic trials (3), ORS formulation (3 comparisons in 2 trials) and 1 diet fiber and 1 lactose-free diet. Only 4 of the trials explicitly described being powered to address diarrhea prevalence or incidence during follow-up [22, 27, 31, 53]. Figure 5 shows the 12 trials (providing 15 estimates due to 3 trials including 3 arms) that reported data on prevalence of diarrhea 7 days or more after presentation (8 zinc [2 of which also assessed vitamin A], 2 ORS (1 of which compared 3 formulations), and 2 probiotic). Only 2 zinc studies [22, 31] and 1 probiotic (*Saccharomyces boulardii*) trial found a reduction in diarrhea prevalence associated with the intervention [41]. The other 6 zinc trials [23, 24, 32–34, 53] and probiotic trial of *Lactobacillus rhamnosus* GG [54] did not find a significant effect on diarrhea prevalence during follow-up. The lactose-free diet reported no effect on the presence of diarrhea at day 12 (*p* = 0.76) but did not report specific prevalences [17]. The 2 trials assessing ORS formulations (providing 3 estimates) did not demonstrate a benefit [34, 55] nor did the 2 vitamin A trials [22, 34].

Of studies reporting on diarrhea frequency indicators other than prevalence of diarrhea at follow-up, findings were heterogeneous. One study found that children given a rice-based diet with green banana or pectin (dietary fiber) were more likely to have recovered from diarrhea by day 5 of follow-up, while most children given the rice-based diet alone continued to have diarrhea until day 10 of follow-up [15]. Another found no children treated with *Lactobacillus reuteri* 17938 to have diarrhea beyond 7-days whereas 17.4% of children without probiotic treatment did have prolonged diarrhea [52]. A trial of 8070 community-based children found that those given zinc with ORS had 2.9 fewer episodes of diarrhea per 100 child-years (95%CI: 0.8, 5.1) than those given ORS alone [27]. A study contrasting 10 day therapeutic zinc (20 mg/day) with 3 months of supplemental zinc (10 mg/day) to the therapeutic zinc course alone found that the long term zinc reduced diarrhea incidence over a 9 month period by 21% (2.05 vs.2.59 episodes / child years) [30]. Compared to children given the multivitamin alone, children given a multivitamin with zinc had an average of 0.33 fewer subsequent diarrhea episodes (95% CI: -0.39, − 0.27) and diarrhea incidence was similarly reduced in the 6 month follow-up period [28, 29]. Conversely, a placebo-controlled trial of therapeutic zinc among 1042 children reported no difference in the mean number of subsequent diarrhea episodes during a 3 month follow-up period nor did 2 smaller zinc trials [29, 32, 48].

## Discussion

While significant progress has been made over the past 25 years in reducing deaths attributed to diarrhea, there is increasing recognition that diarrhea is associated with mortality, subsequent morbidities, and malnutrition in the period after a diarrheal episode [56, 57]. These post-acute sequelae highlight the need, and opportunity, to identify interventions to reduce morbidity and mortality among children presenting with diarrhea. This systematic review appraised diarrhea intervention trials for evidence of effects on post-acute sequelae of diarrhea, including mortality, nutritional status, and diarrhea presence during an extended follow-up period.

We found very few trials that evaluated post-acute diarrheal mortality, and only 1 (of zinc) was explicitly powered to address mortality and found mortality benefit [27]. The other zinc trials did not report a mortality benefit. As summarized in a recent Cochrane review, zinc appears to reduce diarrhea duration, particularly in malnourished children, although the degree to which this effect translates to mortality benefit remains unknown [58]. Therapeutic zinc also appears to have limited to no efficacy on morbidity or growth in children under 6 months of age [53, 59].

Post-acute mortality was assessed in 2 trials of antibiotics that found no mortality benefit, yet were underpowered to do so. Both trials included less than 100 children and only 1 was placebo-controlled. The role of antibiotics in diarrhea management remains controversial. In the absence of diagnostics, diarrhea management guidelines recommend antibiotics only for dysentery or suspected cholera [60, 61]. Limiting antibiotics to these 2 indications may miss other serious enteric infections amenable to antibiotics [62, 63]. In practice however, many children without these indications are treated with an antibiotic, the benefits of which are not well understood [64]. Large placebo-controlled clinical trials are needed to determine the potential harm and/or benefit of antibiotics to reduce post-acute diarrhea morbidity and mortality.

Over 30 trials reported on growth outcomes. Dietary supplementation with macro- or micro-nutrients, high protein and lactose-free diets, and probiotics were assessed for effects on growth with mixed results. We found substantial variability in how growth outcomes were evaluated, making comparisons between studies challenging. Two of the 5 trials of dietary interventions found beneficial impacts on WAZ/WHZ with a high protein isocaloric diet. In a single study, glutamine demonstrated a signal of benefit at 1 time point which was not sustained. Most trials that assessed weight reported no intervention effect; perhaps because weight gain restored through hydration during the acute phase of diarrhea overshadowed weight gain from trialed interventions. High protein diets, either alone or in combination with micronutrients such as zinc, had a modest impact on short to medium term linear growth (3 weeks to 9 months). However, this effect was inconsistently demonstrated. High protein diets may restore the protein loss that can occur during and immediately after infection [65, 66]. Replacing protein may modify growth consequences of diarrhea by increasing protein availability or by influencing hormonal regulation [67–69]. The combination of high protein and zinc may restore integrity of damaged mucosal surfaces and improve nutrient absorption [70–72]. However evidence around the effect of zinc on markers of intestinal permeability, as measured by the lactulose to mannitol ratio, are inconsistent [49, 73, 74]. Specific amino acids may also be important; glutamine has been shown to protect against bacterial translocation through maintenance of the gut barrier in animal models [75–77].

Diarrhea during follow-up was the most commonly reported outcome assessed in this review. Numerous systematic reviews of therapeutic zinc on diarrheal outcomes have been conducted, all of which suggest some benefit [58, 78–80]. The effects of zinc on diarrhea at a specified day of follow-up were recently summarized in a Cochrane review and pooled relative risks of diarrhea at day 3, day 5, and day 7 associated with zinc all showed a statistically significant benefit [58]. Our review included diarrhea assessed at 7 days and beyond (7 days to 4 months) and found inconsistent results, perhaps demonstrating a waning in effect or sub-optimal statistical power at longer follow-up time points. Given therapeutic zinc is recommended for 14 days in current WHO management guidelines yet the data on benefit seems most pronounced within the first 7 days, days 7–14 of the currently recommended zinc course may need further evaluation.

Three of the 4 probiotic trials evaluating diarrhea outcomes demonstrated a benefit on diarrhea during follow-up (*Saccharomyces boulardii, Lactobacillus reuteri* 17938 and 1 of the 2 *Lactobacillus rhamnosus* GG trials*)*. However the *Lactobacillus rhamnosus* GG trial that did not find a benefit in diarrhea during follow-up did report improvements in intestinal function (as measured by the lactulose to mannitol test) and higher immunoglobulin G (IgG) in the subgroup of children with rotavirus infection treated with the probiotic [54]. Most clinical trials of probiotics have been conducted in high-resource settings and have treated and followed children for less than 7 days [81, 82]. Although not included in this review because it was published after the search was conducted, a recent pilot study (n=76) conducted in Botswana found a greater increase in HAZ and reduced diarrhea recurrence over 60-days of follow-up among admitted children with diarrhea randomized to *Lactobacillus reuteri* 17938, [62]. The European Society for Pediatric Gastroenterology recently recommended probiotics, specifically *Saccharomyces boulardii* or *Lactobacillus rhamnosus* GG*,* to reduce the duration and intensity of gastroenteritis [83]. The mechanisms by which probiotics may decrease diarrheal symptoms are largely unknown, but may act by out-competing pathogenic enteric infections for nutrients, restoring gut barrier functions, and/or by restoring gut microbial balance.

This review had several limitations. Most trials, particularly trials used to evaluate mortality, were underpowered. A 2-armed clinical trial powered to detect a 50% reduction in a 3-month diarrheal case fatality rate of 2% would require over 4000 participants, a sample size far larger than most trials reporting mortality outcomes in this review and far smaller than sample sizes required to detect smaller intervention effects or lower case fatality rates. Many trials were excluded because of short length of follow-up and included trials had follow-up times ranging from 7-days to 9 months which could explain heterogeneity between studies, particularly studies of growth outcomes. Some interventions were more represented than others based on available clinical trial data. Markers of enteric function were not included in this review as pre-specified outcomes, despite a growing body of evidence suggesting that enteric dysfunction is linked to poor outcomes following acute illness [84]. Because of the heterogeneity in interventions, outcome measurements, and follow-up time, we did not calculate pooled measures of effect. For the same reason, we did not report a GRADE score for every individual result but rather for overall study quality, and most trials were graded as low or very low. Standardized measures of the nutritional consequences of diarrhea and diarrhea morbidity will be important to enable future meta-analyses. This review includes data from a wide range of geographic, demographic and epidemiologic settings. However, most included trials were conducted in Asia, with less than 15% of all included trials conducted in sub-Saharan Africa (SSA). Diarrhea-mortality rates are higher in SSA than in South Asia and recent projections of childhood mortality into 2030 predict that SSA will contribute to 60% of all childhood deaths [5, 57]. Host characteristics, such as nutritional status and HIV-infection/−exposure vary greatly between these regions making generalizability of intervention effect challenging [85–87].

## Conclusions

In many resource-limited settings, diarrheal episodes in young children are frequent and are associated with increased risk of mortality as well as growth failure and risk of subsequent infections. The mechanisms by which diarrhea and underlying enteric infections lead to morbidity, malnutrition, and mortality are multifactorial, likely requiring multiple complementary interventions to reduce likelihood of recurrence or persistence, promote healing of the gut mucosa, and to replenish lost protein and nutrients. Well-designed, multi-factorial, clinical trials evaluating the extended impact of diarrhea management interventions are urgently needed to reduce the long-term risks associated with diarrhea.

## Additional file

Additional file 1: Supplement. (DOCX 129 kb)

## Abbreviations

CI: Confidence Interval
GRADE: Grading of Recommendations Assessment Development and Evaluation
HAZ: Height-for-age z-score
LAZ: Length-for-age z-score
LMICs: Low- and middle-income countries
MSD: Moderate-to-severe diarrhea
MUAC: Mid upper arm circumference
PR: Prevalence Ratio
RCT: Randomized controlled trial
RR: Relative Risk
RUTF: Ready-to-use therapeutic foods
WAZ: Weight-for-age z-score
WHZ: Weight-for-height z-score
WLZ: Weight-for-length z-score
