# Supplementary material for: Interventions to reduce post-acute consequences of diarrheal disease in children: a systematic review
Source: BMC Public Health. 2018 Feb 1;18:208. doi: 10.1186/s12889-018-5092-7 (PMC5796301; doi:10.1186/s12889-018-5092-7)
Supplement: Additional file 1: — Supplement. (DOCX 129 kb) [file 12889_2018_5092_MOESM1_ESM.docx]

**SUPPLEMENT**

**Methods**

Data were abstracted from included studies, and the following calculations were performed on applicable data. If three or more studies reported data on an outcome using the same metric, or reported data from which a consistent metric could be calculated, forest plots were created to display these results. When a study presented data on relevant outcomes at more than one time point during follow up, the estimates reported closest to the end of follow up were included in the forest plot. When a manuscript presented results as a study group mean with standard deviation, the following equation was used to calculate 95% confidence intervals if not presented in the manuscript: $\bar{x} \pm(1.96)(s/\sqrt{n})$ where $\bar{x}$ is the group mean presented, $s$ is the group standard deviation presented, and $n$ is the group sample size. To calculate an estimate of the difference between group means and the 95% confidence interval for that difference in means, the following equation was used: $\left( \bar{x}_{1}-\bar{x}_{2} \right)\pm\left( 1.96 \right)\sqrt{\left( {s_{1}^{2}}/{n_{1}} \right)+\left( {s_{2}^{2}}/{n_{2}} \right)}$, $\bar{x}$ is the mean presented for each of the two study groups (the intervention and control groups), $s$ is the standard deviation presented for each of the two study groups, and $n$ is the sample size for each of the two study groups. If only the within-group difference in means (mean at follow up compared to mean at enrollment) with a 95% confidence interval were presented, the following formula was used to calculated group standard deviation in order to use the equation above: $\sqrt{n} \times(upper limit-lower limit)/3.92$.[8] If only standard errors of the mean were presented, the following equation was used to calculate standard deviations in order to use the equation above: $SE \times\sqrt{n}$.[8] When a manuscript presented data on proportion or number of patients who had an event of interest (death or diarrhea during follow up), relative risks/prevalence ratios were estimated and 95% confidence intervals calculated assuming a binomial distribution. When a stratum contained a zero value, risk differences were estimated instead of relative risks/prevalence ratios. All calculations were done in Stata 14 IC.
